# Distribution patterns, conservation status and suitable habitat areas of highly valuable medicinal plants in China

**DOI:** 10.3389/fpls.2025.1657997

**Published:** 2025-12-03

**Authors:** Xiuli Gao, Jiejing Gao, Sudhindra R. Gadagkar, Ruyu Yao, Yiheng Li, Sanling Jin, Buhe, Jianhe Wei, Bengang Zhang, Shengxiang Yu, Zhirong Zheng, Yaodong Qi

**Affiliations:** 1 State Environmental Protection Key Laboratory of Regional Eco-Process and Function Assessment, Chinese Research Academy of Environmental Sciences, Beijing, China; 2 Key Laboratory of Bioactive Substances and Resources Utilization of Chinese Herbal Medicine, Ministry of Education & National Engineering Laboratory for Breeding of Endangered Medicinal Materials Institute of Medicinal Plant Development, Chinese Academy of Medical Sciences and Peking Union Medical College, Beijing, China; 3 School of Life Science, Guizhou Normal University, Guiyang, China; 4 Biomedical Sciences Program, College of Graduate Studies, Midwestern University, Glendale, AZ, United States; 5 College of Veterinary Medicine, Midwestern University, Glendale, AZ, United States; 6 Arizona College of Osteopathic Medicine, Midwestern University, Glendale, AZ, United States; 7 Kunming Institute of Botany, Chinese Academy of Sciences, Kunming, China; 8 Research Center for Eco-Environmental Sciences, Chinese Academy of Sciences, Beijing, China; 9 College of Grassland Science and Technology, China Agricultural University, Beijing, China; 10 Forestry and Grassland Bureau of Ewenke Banner, Hulunbuir, Inner Mongolia, China; 11 Department of Life Sciences, Natural History Museum of China, Beijing, China; 12 State Key Laboratory of Plant Diversity and Specialty Crops, Institute of Botany, Chinese Academy of Sciences, Beijing, China; 13 College of Life Science, University of Chinese Academy of Sciences, Beijing, China; 14 China National Botanical Garden, Beijing, China

**Keywords:** medicinal plants, complementarity, weighted endemism, diversity hotspots, conservation status, suitable habitat areas

## Abstract

Medicinal plants represent a critical component of biodiversity. This study focuses on the most valuable species listed in the *Red Data Book of Chinese Medicinal Plants* (RCMPs) to analyze their distribution patterns, identify diversity hotspots, assess conservation status, and predict suitable habitats across China. Results reveal that RCMPs are primarily concentrated in southwestern China, particularly the Qilian Mountains, Qinling-Bashan Mountains, and southeastern Xizang. As the hotspot threshold increases, the conservation effectiveness of provincial nature reserves is surpassed by that of national reserves. Concurrently, overall effectiveness declines, with over 16.43% of hotspot grids (containing >91.48% of species) remaining unprotected, indicating persistent conservation gaps in southwestern China and Xinjiang. Predictive modeling suggests a potential decrease in RCMPs richness in southeastern and central China. Under the RCP 2.6 and 8.5 scenarios for 2070, approximately 42.99% and 57.01% of RCMPs are projected to experience habitat contraction and expansion, respectively. Based on these findings, we propose a comprehensive priority conservation framework to inform future protection planning and promote the long-term conservation and sustainable use of China’s medicinal plants.

## 1 Introduction

Medicinal plants serve as a valuable source to meet lots of people’s healthcare needs (Pei and Huai, 2007; World Health Organization, 2013; Hamilton, 2004; Chen et al., 2016; Xia et al., 2022; Shan et al., 2023). With increasing health awareness, the use of medicinal plants is rapidly growing worldwide (Srivastava, 2000; Chen et al., 2016). Human livelihood in parts of the world is also dependent on medicinal plants and their conservation (Hamilton, 2004). The 10th Conference of the Parties to the Convention on International Trade in Endangered Species of Wild Fauna and Flora (CITES) passed a resolution specifically calling for attention on species used in traditional medicine that are threatened with extinction and for effective protection measures to be taken (Wijnstekers, 2018).

Successful conservation of biodiversity by means of identifying diversity hotspots and conservation gaps is crucial, especially in the current context of limited resource availability (Yan et al., 2013; Huang et al., 2016; Shrestha and Wang, 2018; Ye et al., 2020; Yang et al., 2022). The species richness algorithm, which is simple and easy to implement, has been widely applied in studies identifying diversity hotspots (Prendergast et al., 1993; Myers et al., 2000; Zhang et al., 2021). On the other hand, complementarity algorithms can help avoid the inclusion of multiple areas with similar species and are increasingly being employed to select priority areas for conservation (Dobson et al., 1997; Chi et al., 2017; Shrestha and Wang, 2018). Similarly, weighted algorithms can be used to prioritize the protection of narrow-range species (Williams et al., 1996; Williams, 2000; Huang et al., 2013; Veach et al., 2017; Qin et al., 2022). In recent years, a trend has developed toward the use of comprehensive approaches for investigating geographical distribution patterns, identifying biodiversity hotspots and finally evaluating the effectiveness and gaps of current conservation measures (Huang et al., 2016; Xue et al., 2023; Qin et al., 2023; Xia et al., 2022; Hu et al., 2024). In addition, other factors must be considered to comprehensively assess the conservation value of biodiversity hotspots, such as the presence of distinct taxonomic groups including threatened and protected species (Forest et al., 2007; Huang JH et al., 2012; Orsenigo et al., 2018; Qin et al., 2023; Xue et al., 2023). Moreover, to achieve better biodiversity conservation, the Aichi targets (top 17%) and the Post-2020 Biodiversity Framework (top 30%) have been proposed, but these measures have not yet been fully implemented (Secretariat of the Convention on Biological Diversity, 2020).

In recent years, climate change has been recognized as an important factor threatening global biodiversity, causing an increase in extinction rates as well as the loss of suitable habitat ranges (Chen et al., 2011; Peng et al., 2022; Ren et al., 2012; Pimm et al., 2014; Xin et al., 2024). The limited geographic distributions and small population sizes of threatened species make them more sensitive to climate change (Huang, 2011; Berry et al., 2013; Garcia et al., 2013; Fortini and Dye, 2017; Xue et al., 2021). To date, many studies have focused on prediction analysis for specific taxa (Hu et al., 2015; Li et al., 2021). Xia et al. (2022) used the MaxEnt model to predict and analyze suitable habitats for 481 threatened medicinal plants in China, revealing a clear south-to-north shift in their potential distribution areas associated with climate change. This shift will result in significant loss of suitable habitats in south China in the future. (Yu et al., 2023) predicted and analyzed the potential distribution of national key protected wild plants and predicted that climate change would impact their distribution. Previous studies have shown that the sensitivity of species to climate change is an essential component of biodiversity conservation planning (Feeley and Silman, 2009; Thomas et al., 2004). To establish a prioritized conservation framework, assessment of changes in the number and distribution of species threatened by future climate changes is urgently needed.

Approximately 3, 000 Chinese herbal medicinal species are currently marketed, with nearly 70% of these species sourced from wild resources (Huang LQ et al., 2012). Overexploitation and loss of habitat pose serious threats to the survival of wild medicinal plants (Yang et al., 2000). Moreover, invasive species, pests, pathogens, and pollution are also reported as seriously threatened factors, which influence the quality and productivity of medicinal plants (Chi et al., 2017; Xia et al., 2022; Shan et al., 2023). Recent studies of medicinal plants have focused on their geographic distribution patterns, diversity hotspots, and conservation effectiveness and gaps (Li et al., 2016; Chi et al., 2017; Xia et al., 2022; Shan et al., 2023; Feng et al., 2023).

However, due to inconsistent taxonomic sampling and research methods and low-resolution geographic distribution information, there is a huge need for additional studies to establish priority conservation areas (PCA) and management strategies for Chinese medicinal plants. For example, (Chi et al., 2017) analyzed the distribution pattern, hotspots, and conservation gaps for 535 threatened medicinal plants at the county level and found that 14.3% of hotspot counties had no protection measures in place. (Xia et al., 2022) studied the geographic distribution patterns and conservation status of over 9, 000 medicinal plants in China based on 50-km resolution grid analysis, identifying three new hotspot regions and significant protection gaps in northwest and southwest China. (Shan et al., 2023) studied the geographic distribution patterns of 2, 700 commonly used medicinal plants at 100-km grid resolution but did not evaluate the effectiveness of their protection measures. Notably, plants listed in the recently published *Red Data Book of Chinese Medicinal Plants* (RCMPs) are medicinal plants with high popularity and significant medicinal value, but with clear threats to their wild populations, unfortunately, representing typical examples of Chinese medicinal plant resources. Therefore, this book is an important resource for priority management of Chinese medicinal plant conservation (Huang et al., 2022). However, in the absence of specific research, the species richness distribution patterns, diversity hotspots, conservation effectiveness and gaps, and impacts of climatic changes on potential suitable habitat areas remain elusive.

Recent studies on conservation estimation from species richness distribution patterns have advocated the use of large distribution datasets and comprehensive approaches to avoid biases generated by limited sampling (Xue et al., 2023; Qin et al., 2023; Yang et al., 2022; Zhang et al., 2022; Xia et al., 2022). Based on over 80, 000 occurrence records with precise coordinates and using comprehensive approaches including the species richness, complementarity, and weighted endemism algorithms as well as different species properties, this study aimed to: (1) identify the geographical distribution patterns and diversity hotspots of RCMPs; (2) evaluate the conservation status of RCMPs and identify conservation gaps in current conservation networks; (3) analyze the potential impacts of climate change on suitable habitat areas of RCMPs; and (4) propose a novel priority conservation framework based on the research findings and targeting the current challenges facing the protection and utilization of RCMPs.

## 2 Materials and methods

### 2.1 Checklist and geographical distribution of RCMPs

The *Red Data Book of Chinese Medicinal Plants* contains 464 of the most valuable and threatened medicinal plants in China (Huang et al., 2022). In this study, six groups were excluded, including fungi, algae, wild extinct species, and species with unclear taxonomic status. The final checklist used for analysis consisted of 458 species, including 264 threatened medicinal plants (covering the categories of critically endangered [CR], endangered [EN], and vulnerable [VU]), 56 near-threatened (NT) species, and 138 species of conservation concern (CC). The list of species is presented in (**Supplementary Table S1**). The concept of endemic species refers to a species that is limited in distribution exclusively to a specific geographic area (Anderson, 1994). Among the 458 species, 232 are endemic to China based on the “*Catalogue of Life China 2022 Annual Checklist*” (The Biodiversity Committee of Chinese Academy of Sciences, 2022) (**Supplementary Table S1**). Geographical distribution information for RCMPs was mainly derived from the distribution information of specimens in the Chinese Virtual Herbarium (http://www.cvh.ac.cn/) and the “Information Database of the Distribution of *Red Data Book of Chinese Medicinal Plants*”, consisting of 86, 210 geographical distribution records. Records lacking detailed collection sites and records with invalid data were excluded.

### 2.2 Geographical distribution patterns and correlation analysis of RCMPs

To assess the geographical distribution pattern of RCMPs, we divided the land area of China into a grid of 4069 cells, with a resolution of 50 km × 50 km, using ArcGIS 10.6 software in accordance with previous studies (Xue et al., 2023; Xia et al., 2022). The geographical distribution patterns of all, endemic, and threatened RCMPs were analyzed using three algorithms, namely species richness, complementarity, and weighted endemism. Species richness is a traditional and commonly used indicator of species diversity hotspots (Orme et al., 2005; Pimm et al., 2014), and this method defines the region with most species as a hotspot based on the number of species present in each cell.

The complementarity algorithm identifies the number of grid cells acting as hotspot areas by selecting the smallest area that maximizes the representation of the biodiversity (Dobson et al., 1997). In the complementarity algorithm, the first step is to select the grid cells with the highest species richness and all the species appearing in those cells are excluded from further consideration. Next, the cell with the largest number of complementary (remaining) species is selected, and this analysis is continued in an iterative manner until all the species in the area are exhausted.

Another metric, the weighted range size rarity, was also calculated using the method of Williams et al. (1996). This is a metric that combines the rarity of species in a given area with the size of their geographic range. Specifically, it assigns different weights to species based on the reciprocal of their distribution area, and the weighted values of all species in a grid cell are summed to obtain the weighted endemism of the cell (Linder, 2001; Huang et al., 2016; Qin et al., 2023).

To detect if the distribution patterns obtained from these three algorithms are correlated, the “corrplot package” in R (version 4.2.1) was used to calculate the Pearson correlation coefficient between each pair of results (Wei and Simko, 2017). The Pearson correlation coefficient (r) was then classified into five categories: negligible correlation (0 < |r| < 0.1), weak correlation (0.10 ≤ |r| < 0.40), moderate correlation (0.40 ≤ |r| < 0.70), strong correlation (0.70 ≤ |r| < 0.90), and very strong correlation (0.90 ≤ |r| < 1) (Schober et al., 2018).

### 2.3 Diversity hotspot identification for RCMPs

The identification of RCMPs diversity hotspots should be based on various biological indicators, including species diversity, species endemism, and the number of threatened species (Myers et al., 2000; Huang et al., 2016, Huang JH et al., 2012; Zhao et al., 2016). In this study, we identified priority conservation areas for RCMPs based on the results of the three algorithms. First, we standardized and summed the richness values for all species, endemic species, and threatened species in each grid using the three algorithms (Xue et al., 2021). Next, we selected the top 5% (140 cells), top 10% (281 cells, with the 280th and 281st cells having equal rank and value), top 17% (476 cells), and top 30% (841 cells, with the 840th and 841st cells having equal standardized sum of richness values) of the cells identified using each algorithm as hotspots and overlaid the results. Finally, we calculated the sum of the order (rank) of each hotspot grid cell in the respective algorithms and sorted the resulting values (Xia et al., 2022). Cells identified by all three algorithms were considered Class-I hotspots, those identified by two algorithms were considered Class-II hotspots, and those identified by only one algorithm were considered Class-III hotspots.

### 2.4 Conservation effectiveness and gaps for RCMPs

Based on the identified diversity hotspots and current conservation networks, we ascertained conservation effectiveness and gaps. The geodatabase of current nature reserves used in this study is based on the list of nature reserves issued by the Ministry of Ecology and Environment of China and the downloaded shapefiles from the World Database on Protected Areas (WDPA, https://protectedplanet.net). The delineation of national nature reserves (NNRs) and provincial nature reserves (PNRs) is clear, strict, and standardized, forming an important cornerstone of biodiversity conservation in China (Zhang et al., 2015; Chi et al., 2017). We focused on evaluating the 464 NNRs and 806 PNRs in terms of conservation effectiveness for RCMP diversity hotspots. The conservation effectiveness and gaps in diversity hotspots and RCMPs were evaluated for three categories of grid cells: those protected by NNRs, by PNRs, and by both types of reserves (hereinafter referred to as NNRs-PNRs). If there is no distribution of nature reserves in a given cell, it is identified as a conservation gap (Huang et al., 2016; Chi et al., 2017; Xie et al., 2022).

### 2.5 Impacts of climate change on the distribution of RCMPs

Many threatened species are highly sensitive to environmental changes, and thus reflect the impact of climate change on biodiversity (Chen et al., 2011). The MaxEnt (Maximum Entropy) model can be used to predict the potential distribution of species under different climate scenarios (Phillips et al., 2006). It offers several advantages, including ease of use, fast operation, small sample size requirements, and high prediction accuracy (Phillips et al., 2006; Zhang et al., 2021; Soilhi et al., 2022). This model has been applied to the study of potential species distribution areas for the purposes of introducing species to new habitats and conducting *ex-situ* conservation research, thereby providing new information for biodiversity conservation. In this study, we used MaxEnt (version 3.4.1) to predict suitable habitats for threatened RCMPs under various environmental conditions. We obtained 19 bioclimatic variables from WorldClim (http://www.worldclim.org) at a resolution of 10 min for the recent past (1960–1990, version 1.4) and future (2070, CMIP5). Future climate variables are based on the BCC-CSM1–1 model (Xin et al., 2013) under the lowest and highest representative concentration pathways (RCP 2.6 and 8.5, respectively). To minimize overfitting, we calculated the possible correlations between 20 environmental variables (Bio1–Bio19 and elevation, **Supplementary Table S2**), and removed one variable from each pair of highly correlated variables (r > 0.85). As a result, ten variables (Bio1, Bio2, Bio3, Bio4, Bio5, Bio12, Bio14, Bio15, Bio18, and elevation) were selected for prediction of potential suitable habitat areas for threatened RCMPs under a changed climate.

Due to the limited predictive ability of MaxEnt for species with less than five distribution points (Pearson et al., 2006; Tang et al., 2018), 36 species (7.86%) were excluded from analysis. As a result, this study focused on the prediction of potential suitable habitat areas for 422 RCMPs (92.14%) with at least five distribution points. For species with 5–29 occurrence records, we used the jackknife approach (Pearson et al., 2006), while for species with more than 30 records, we used the cross-validation approach (Tang et al., 2018) to evaluate the robustness of the model. We used 75% of the distribution data as training data and the remaining 25% as testing data, with default settings. After prediction of the suitable habitats for each medicinal plant, we used ArcGIS 10.6 to extract suitability values for each plant in each grid. During this process, we first excluded species with an area under the receiver operating characteristic curve (AUC) value less than 0.7 (Swets, 1988), and then removed data with suitability values less than 0.6, as suitability values greater than 0.6 indicate high potential for a species to be present in the environment (Yang et al., 2013; Yan et al., 2020). Therefore, we assumed that a species occurs in a grid cell if its suitability value in the cell is > 0.6. In this manner, we could predict potential species richness under current and future climate scenarios, as well as potential changes in the geographic distribution of species richness. Additionally, we calculated changes in the suitable habitat area available for predicted species in the future (decrease, no change, or increase), as well as changes in the threatened categories of unthreatened RCMPs under climate impacts based on the loss of suitable habitat area (LSA) as follows: EX, LSA = 100%; CR, 80% ≤ LSA < 100%; EN, 50% ≤ LSA < 80%; VU, 30% ≤ LSA < 50% (Peng et al., 2022); and CC, LSA < 30% (Huang et al., 2022).

## 3 Results

### 3.1 Geographical distribution patterns of RCMPs

**Figure 1:**
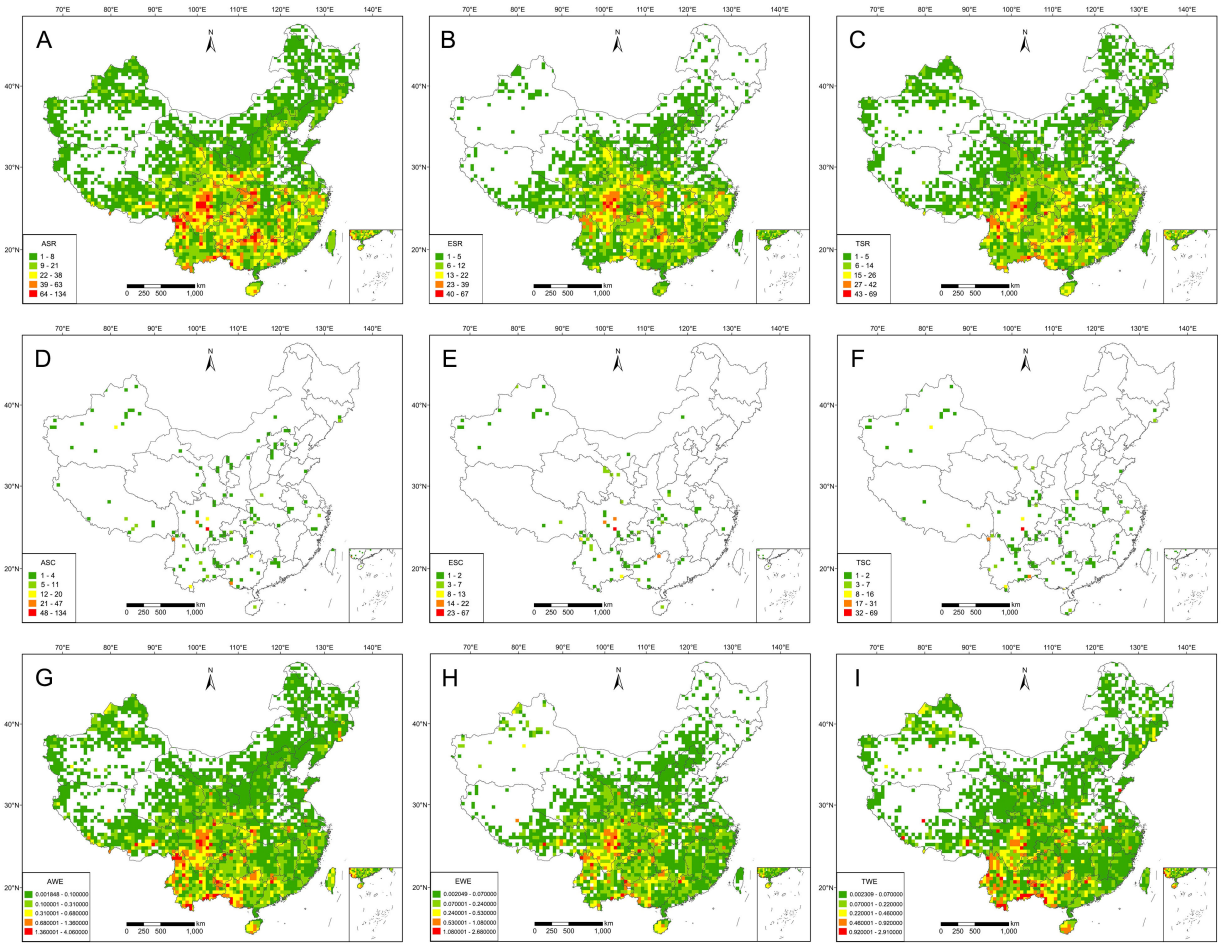
Geographical distribution patterns of Red Data Book of Chinese Medicinal Plants (RCMPs). **(A)** Species richness of all RCMPs (ASR). **(B)** Species richness of endemic RCMPs (ESR). **(C)** Species richness of threatened RCMPs (TSR). **(D)** Species complementarity of all RCMPs (ASC). **(E)** Species complementarity of endemic RCMPs (ESC). **(F)** Species complementarity of threatened RCMPs (TSC). **(G)** Weighted endemism of all RCMPs (AWE). **(H)** Weighted endemism of endemic RCMPs (EWE). **(I)** Weighted endemism of threatened RCMPs (TWE).

Based on the species richness algorithm, most RCMPs are distributed mainly in the mountain ranges of central and southwestern China, including the Hengduan Mountains, the Bashan-Wushan Mountains, the Sino-Vietnamese border, and the Nanling Mountains (**Figure 1A**; **Supplementary Figure S1**). The geographical distribution patterns of endemic RCMPs and threatened RCMPs are congruent, indicating a distribution mainly in the Hengduan Mountains, Wushan Mountains, and Nanling Mountains in southwestern and central China (**Figures 1B, C**). The complementarity algorithm shows a similar but more dispersed distribution pattern for all species, including endemic and endangered species, with the highest diversity generally observed in the Hengduan Mountains of Sichuan (**Figures 1D–F**). The results of the weighted endemism algorithm are similar to those of the species richness algorithm, but grid cells with high levels of weighted endemism are more prominent in southwest China (**Figures 1G–I**). Correlation analysis of the three algorithms shows that the distribution patterns obtained using a single algorithm are highly correlated, whereas a moderate correlation exists between the species richness algorithm and the weighted endemism algorithm (r = 0.71–0.84, p < 0.01) (**Supplementary Figure S2**), and the correlations of the complementarity algorithm with the species richness algorithm and the weighted endemism algorithm are statistically significant but weak (r = 0.25–0.3, r = 0.35–0.45, respectively; p < 0.01) (**Supplementary Figure S2**).

### 3.2 Diversity hotspots for RCMPs

**Figure 2:**
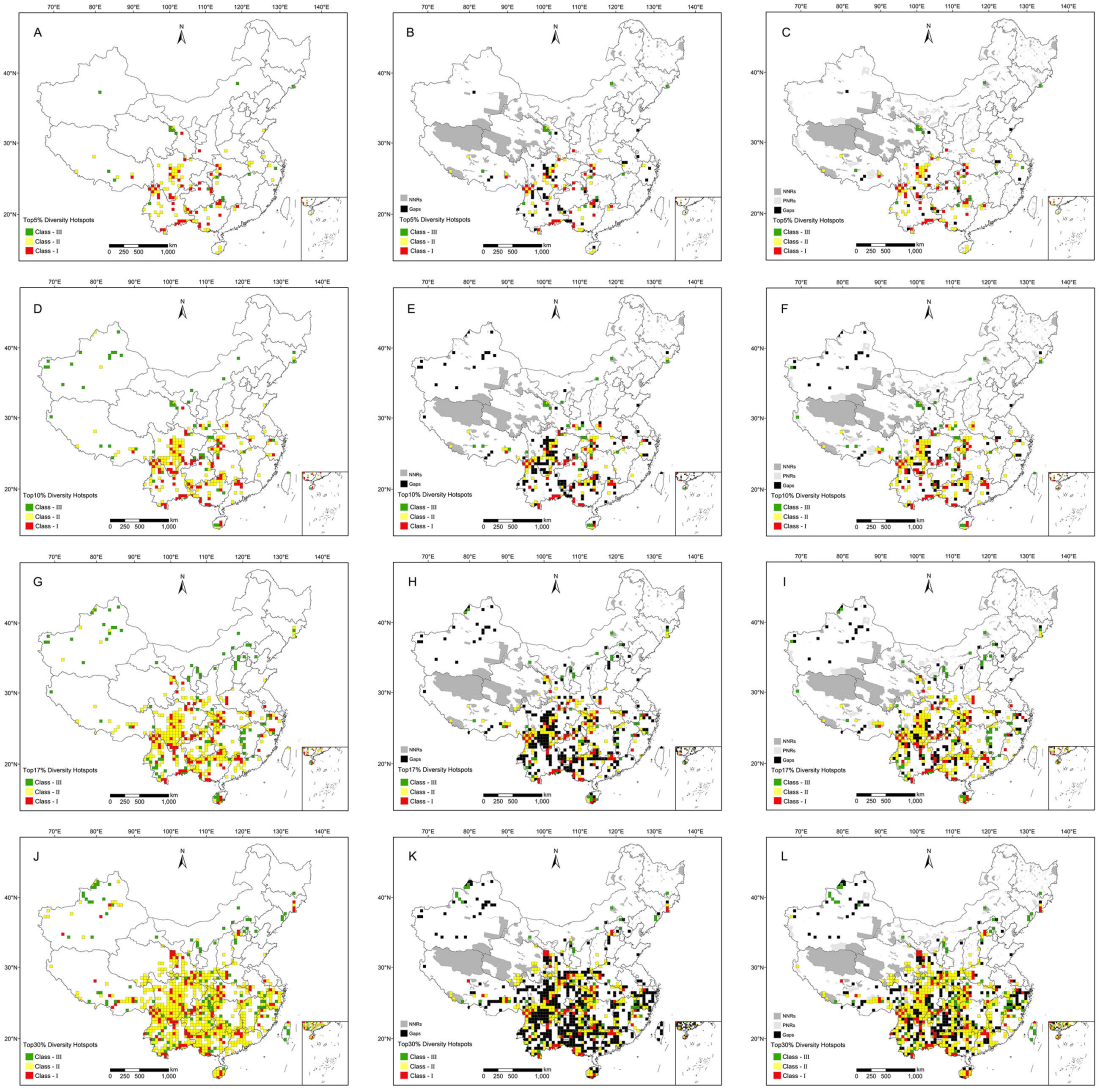
Diversity hotspots for Red Data Book of Chinese Medicinal Plants (RCMPs). **(A)** Top 5% hotspots. **(B)** Conservation gaps of top 5% hotspots under national nature reserves (NNRs). **(C)** Conservation gap of top 5% hotspots under NNRs and provincial nature reserves (PNRs). **(D)** Top 10% hotspots. **(E)** Conservation gaps of top 10% hotspots under NNRs. **(F)** Conservation gap of top 10% hotspots under NNRs and PNRs. **(G)** Top 17% hotspots. **(H)** Conservation gaps of top 17% hotspots under NNRs. **(I)** Conservation gap of top 17% hotspots under NNRs and PNRs. **(J)** Top 30% hotspots. **(K)** Conservation gaps of top 30% hotspots under NNRs. **(L)** Conservation gap of top 30% hotspots under NNRs and PNRs.

Based on four different thresholds, hotspots are mainly distributed in south China, with a few located in north China; these regions include at least 446 (97.38%) RCMPs (**Table 1**). Based on the threshold of top 5%, 140 grid cells were identified as final hotspots for RCMPs, with these hotspot grids distributed mainly in the Hengduan Mountains, the junction of northern Yunnan and southern Sichuan, the Bashan-Wushan Mountains in central China, the Nanling Mountains, and the Sino-Vietnamese border (**Figure 2A**). The top 10% of cells includes 281 hotspot cells; in addition to the top 5% hotspots, hotspot cells located in the eastern Dabie Mountains, Tianmu Mountains, Yandang Mountains, and Hainan Island are also included (**Figure 2D**). The top 17% hotspot results include 476 hotspot cells, which cover most of the top 10% areas, showing a significant increase in hotspot grid area and the addition of the Wuyi Mountains as a hotspot (**Figure 2G**). The top 30% hotspot results include 841 hotspot cells covering most of the top 17% areas, with a significant increase in hotspot grid density in the Tianshan Mountains, Changbai Mountains, northern Taihang Mountains, and eastern China (**Figure 2J**).

**Table 1:** Analysis and statistical evaluation of conservation effectiveness and gaps in the protection of all species, endemic species, threatened species, and final hotspots.

Grid cell type	Taxa	Top 5%	Top 10%	Top 17%	Top 30%
Final hotspots	Grid cells	140	281	476	841
	All Species	446/97.38%	458/100.00%	458/100.00%	458/100.00%
	Endemic Species	230/99.14%	232/100.00%	232/100.00%	232/100.00%
	Threatened Species	258/97.73%	264/100.00%	264/100.00%	264/100.00%
Grid cells protected by NNRs	Grid cells	90/64.29%	156/55.52%	238/50.00%	374/44.47%
	All Species	416/90.83%	426/93.01%	434/94.76%	439/95.85%
	Endemic Species	216/93.10%	218/93.97%	221/95.26%	221/95.26%
	Threatened Species	238/90.15%	243/92.05%	250/94.70%	252/95.45%
Grid cells protected by PNRs	Grid cells	73/52.14%	136/48.4%	242/50.84%	400/47.56%
	All Species	381/83.19%	409/89.30%	424/92.58%	434/94.76%
	Endemic Species	204/87.93%	213/91.81%	215/92.67%	218/93.97%
	Threatened Species	214/81.06%	231/87.50%	240/90.91%	245/92.80%
Grid cells protected by NNRs- PNRs	Grid cells	117/83.57%	213/75.8%	356/74.79%	590/70.15%
	All Species	428/93.45%	441/96.29%	447/97.60%	450/98.25%
	Endemic Species	222/95.69%	225/96.98%	227/97.84%	227/97.84%
	Threatened Species	244/92.42%	250/94.70%	256/96.97%	258/97.73%
Grid cells unprotected by NNRs	Grid cells	50/35.71%	125/44.48%	238/50.00%	467/55.53%
	All Species	381/83.19%	426/93.01%	437/95.41%	440/96.07%
	Endemic Species	216/93.10%	217/93.53%	218/93.97%	220/94.83%
	Threatened Species	238/90.15%	239/90.53%	247/93.56%	249/94.32%
Grid cells unprotected by PNRs	Grid cells	67/47.86%	145/51.6%	234/49.16%	441/52.44%
	All Species	409/89.30%	429/93.67%	434/94.76%	438/95.63%
	Endemic Species	206/88.79%	216/93.10%	217/93.53%	219/94.40%
	Threatened Species	214/81.06%	248/93.94%	250/94.70%	252/95.45%
Grid cells unprotected by NNRs-PNRs	Grid cells	23/16.43%	68/24.2%	120/25.21%	251/29.85%
	All Species	325/70.96%	380/82.97%	409/89.30%	419/91.48%
	Endemic Species	158/68.10%	187/80.60%	199/85.78%	207/89.22%
	Threatened Species	185/70.08%	215/81.44%	230/87.12%	237/89.77%

The denominator of all proportions is the total number of corresponding groups; NNRs (National Nature Reserves) and PNRs (Provincial Nature Reserves).

Based on China’s natural geography, the hotspot cells identified with these four thresholds were divided into 21 hotspot regions (**Supplementary Figure S3**), including (1) Qilian Mountains, (2) Qinling-Bashan Mountains, (3) southeastern Xizang, (4) southern Hengduan Mountains, (5) northern Hengduan Mountains, (6) Xishuangbanna, (7) the junction of northern Yunnan and southern Sichuan, (8) Sino-Vietnamese border, (9) the junction of southern Guizhou and northwestern Guangxi, (10) Nanling Mountains, (11) Dalou -Wuling Mountains, (12) Wushan Mountains, (13) Hainan Island, (14) eastern Qinling Mountains, (15) Dabie Mountains, (16) Tianmu Mountains, (17) Yandang Mountains, (18) Wuyi Mountains, (19) Tianshan Mountains, (20) Changbai Mountains, and (21) northern Taihang Mountains (**Supplementary Figures S3A–D**). These hotspot regions are mainly distributed in southwestern China, with a few in northern China. Hotspots 1–12 were identified using all four thresholds, and the top 10% threshold identified five additional areas, designated hotspots 13, 14, 15, 16, and 17 (**Supplementary Figure S3B**). Furthermore, the top 17% threshold contributed hotspot 18 (**Supplementary Figure S3C**), and analysis of the top 30% identified hotspots 19, 20, and 21 (**Supplementary Figure S3D**).

### 3.3 Conservation effectiveness and gaps of RCMPs

Based on the analysis of the conservation effectiveness for four hotspot thresholds – top 5%, top 10%, top 17%, and top 30%. NNRs protected more than 64.29% (90/140) of hotspot cells (**Table 1**), while PNRs protected more than 52.14% (73/140) of them (**Table 1**). With NNRs-PNRs, more than 83.57% (117/140) of such cells were effectively protected (**Table 1**). Thus, the joint conservation effectiveness of NNRs and PNRs is much higher than the effectiveness of either type alone. However, compared to the conservation effectiveness of PNRs, NNRs showed a sharper decrease in conservation effectiveness with increasing hotspot threshold (from top 5% to top 30%, **Supplementary Figure S4**). At the species level, the current conservation networks of NNRs, PNRs, and NNRs-PNRs protected at least 90.83%, 83.19%, and 93.45% of RCMPs, and up to 95.85%, 94.76%, and 98.25% of RCMPs, respectively, regardless of hotspot threshold (**Supplementary Figure S5**, **Table 1**).

Conservation gap analysis showed that at least 35.71% (50 out of 140, top 5%) of hotspot grid cells are unprotected by NNRs, most of which are distributed in hotspots of the Hengdaun Mountains, the junction of southern Guizhou and northwestern Guangxi, Nanling Mountains and Xinjiang (**Figure 2B**). PNRs cover some conservation gaps of NNRs in key hotspot areas, including Hengduan Mountains, western Guangxi, southwestern Guangdong and Nanling Mountains. In the conservation gaps of NNRs-PNRs, at least 16.43% (23 out of 140, top 5%) of hotspot cells were not effectively protected (**Table 1**). Most of these conservation gaps were scattered in southwest China and Xinjiang (**Figures 2C, F, I, L**). Conservation gaps were always more abundant in northern Xinjiang and Guizhou, regardless of the hotspot threshold used (**Figures 2 C, F, I, L**). At the species level, these conservation gaps contained at least 70.96% of RCMPs, including 68.10% of endemic and 70.08% of threatened species (**Supplementary Table S1**). In addition, at the population level, 13 (2.8%) species had less than 30% of their occurrence records covered by NNRs, PNRs, or both, whereas 42 (9.17%) species received effective conservation across more than 90% of their occurrence records (**Supplementary Figure S6**; **Supplementary Table S1**).

### 3.4 Response of RCMPs to climate change

**Figure 3:**
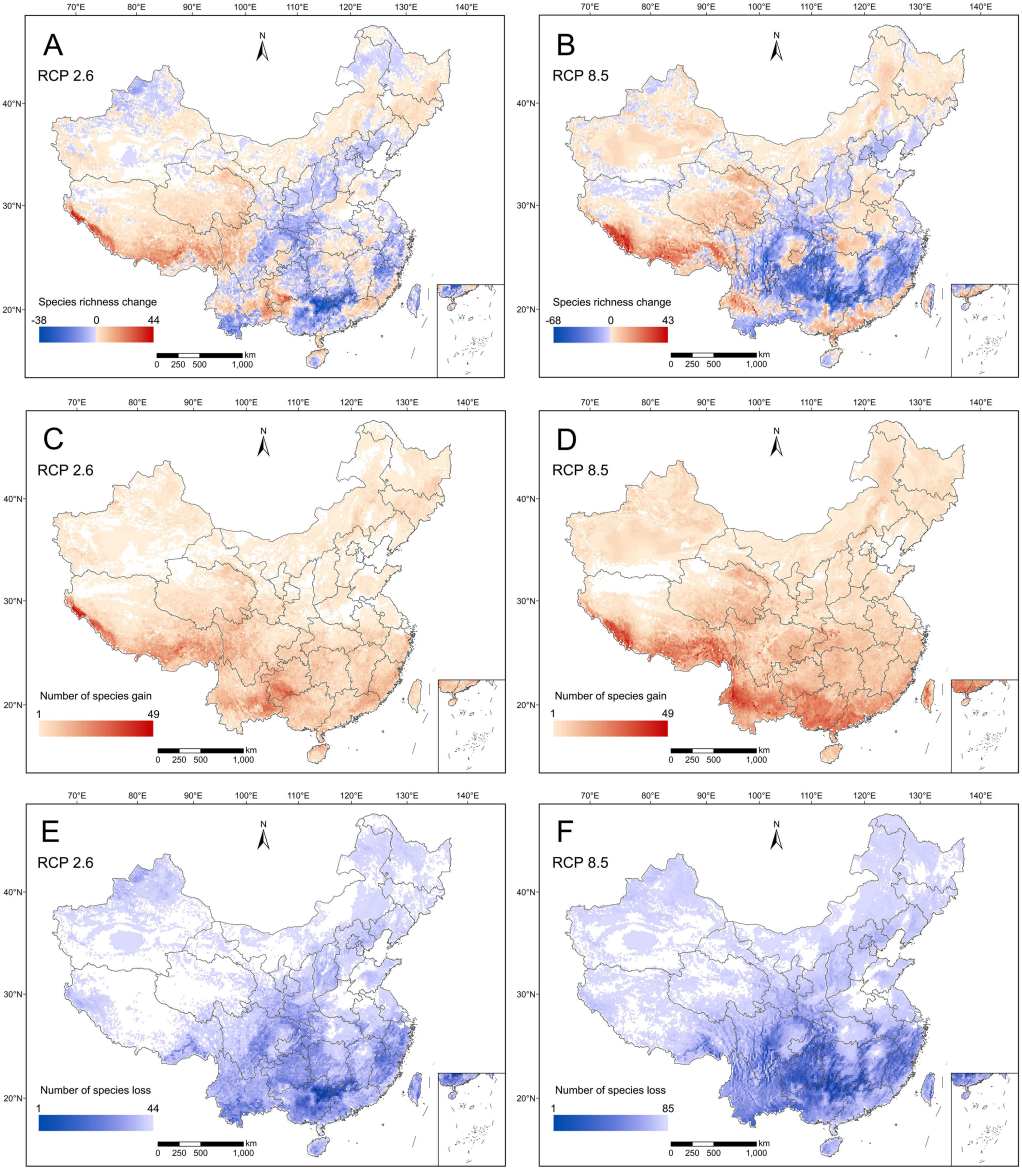
Species richness change **(A, B)**, number of species gain **(C, D)**, and number of species loss **(E, F)** for Red Data Book of Chinese Medicinal Plants (RCMPs) in China by 2070 under two emission scenarios, representative concentration pathway [RCP] 2.6 and RCP 8.5.

In total, 422 RCMPs (92.14% of all RCMPs included in this study) were used for niche modeling, of which 421 species had test AUC values of > 0.7. Furthermore, 88.86% of modeled species had test AUC values of > 0.9, a high level of accuracy for modeling RCMPs (**Supplementary Table S3**). Predictive analysis of potential distribution areas showed that potential suitable habitats are mainly distributed in southwest China, central China and Hainan under current and future climate scenarios (**Supplementary Figure S7**). However, considerable changes in species richness were observed in southeast and central China (**Figures 3A, B**). Compared to the current climate scenario, the species richness of RCMPs was predicted to decrease in some areas under the future RCP 2.6 climate scenario, including the Bashan-Wushan Mountains, Xishuangbanna, the Dalou Mountains, Wuling Mountains, Luoxiao Mountains, Nanling Mountains, and western Fujian in central and southeast China (**Figures 3A, E**). Under the future RCP 8.5 climate scenario, the same areas experienced more significant decreases in species richness (**Figures 3B, F**). Interestingly, southeast Xizang and the central Himalayas would potentially gain the most species among regions (**Figures 3A–D**).

Statistical analysis of the shift in habitat areas with suitability values greater than 0.6 showed that, compared with the RCP 8.5 climatic scenario, 42.99% (vs. 56.06%) of RCMPs will be faced with habitat contraction in 2070 under the RCP 2.6 climatic scenario, while 57.01% (vs. 43.94%) of RCMPs are expected to experience habitat expansion (**Supplementary Figure S8**; **Supplementary Table S3**). When comparing potential suitable habitat areas under present and future climate scenarios, a much clearer shift in potential suitable habitat areas is obtained under the RCP 8.5 climate scenario than RCP 2.6. Compared with RCP 2.6, a much larger proportion of RCMPs (35.87% vs. 9.03%) will experience habitat shifts (expansion or contraction), covering over 60% of potential suitable habitat areas under the RCP 8.5 climate scenario. In terms of the loss of potential suitable habitat areas, under the RCP 2.6 scenario, only 1.19% of RCMPs are predicted to lose over 60% of potential suitable habitat areas, while this proportion increased to 20.43% under the RCP 8.5 scenario, with two species losing all their suitable habitats (**Supplementary Figure S8**).

Additionally, in accordance with the shift in potential suitable habitat areas between current and future climate scenarios, evaluation of the change in threatened categories for unthreatened RCMPs (NT and CC categories). The results showed that, in 2070 under the RCP 2.6 scenario, 5 (1.09%) and 13 (2.84%) species were predicted to be upgraded to the EN and VU categories, respectively, while under the RCP 8.5 scenario, 15 (3.28%), 32 (6.99%), and 25 (5.46%) species were predicted be upgraded to the CR, EN, and VU categories, respectively (**Table 2**). Of these species, 18 (3.93%) were predicted to become threatened under any climate scenario (**Supplementary Table S4**).

**Table 2:** Statistical analysis of changes in the threatened categories of unthreatened RCMPs with climate change.

Current threatened categories	CC			NT		
Categories	CR	EN	VU	CR	EN	VU
RCP 2.6 threatened categories	0/0.00%	3/0.66%	8/1.75%	0/0.00%	2/0.44%	5/1.09%
RCP 8.5 threatened categories	9/1.97%	17/3.71%	21/4.59%	6/1.31%	15/3.28%	4/0.87%

CC, conservation concern; NT, near threatened; CR, critically endangered; EN, endangered; VU, vulnerable.

## 4 Discussion

Given the high conservation value of RCMPs, we investigated their geographic distribution patterns and diversity hotspots, evaluated conservation effectiveness and gaps in the current conservation networks. Secondly, we predicted future potential suitable habitat areas, and then we analyzed shifts of suitable habitat areas as well as climate impacts on threatened categories. This study provides accurate and important data for prioritized management of RCMPs through various conservation strategies and serves as a reference for the conservation and prioritized management of other plants of high economic value.

### 4.1 Geographical distribution pattern of RCMPs

Based on large-scale and high-resolution distribution data, this study explores the geographic distribution patterns of species richness, species complementarity, and weighted endemism of RCMPs, using these measures to identify the areas with the highest diversity of RCMPs. Such areas are located in the mountainous regions of southern and southwestern China, including the Hengduan Mountains, Bashan Mountains, Wushan Mountains, Nanling Mountains, and the Sino-Vietnamese border, which is congruent with the distribution patterns of all medicinal plants (Xia et al., 2022) and threatened higher plants (Xue et al., 2023). This similarity may arise because the mountainous climate, soil, and elevation of this region provide varied habitat types (Tang et al., 2006; Qin et al., 2023), driving the diversity distribution patterns of RCMPs. However, many zones have high population density and abundant human activities (Xu et al., 2019; Su et al., 2020) that pose risks to RCMPs.

Comparing the RCMP distribution patterns among the three algorithms listed above, the species richness algorithm and the weighted endemism algorithm generated similar spatial distribution patterns (**Figure 1**). However, the distribution patterns of the species richness algorithm for all, endemic, and threatened species are mainly distributed in Bashan and Wushan in central China, whereas the distribution patterns of the weighted endemism algorithm for all, endemic, and threatened species are mainly located in southwestern China, namely the Hengduan Mountains, southern Yunnan, and southern Guangxi. This difference indicates that more narrow-range RCMPs are distributed in southwestern China, indicating a more urgent need for conservation efforts in these areas. The distribution patterns of the complementarity algorithm for all, endemic, and threatened species are more scattered overall and are inconsistent with the patterns generated from the other two algorithms. This inconsistency is largely driven by the exclusivity of the complementarity algorithm. Although the species richness and weighted endemism algorithms are strongly correlated, the weighted endemism algorithm emphasizes narrow-range species with high weighting values (Huang et al., 2016; Yu et al., 2017; Xue et al., 2023), while the complementarity algorithm selects the minimal area containing the maximum number of species (Shrestha and Wang, 2018). The inconsistency among the geographical distribution patterns generated using various algorithms can provide information to support targeted conservation strategies for specific groups such as endemic species, regional representative species, and narrow-range species. Therefore, in future hotspot analyses, multiple algorithms and multiple biological attribute indicators should be considered to achieve systematic conservation planning, using the most effective method of biodiversity conservation, which is also relatively comprehensive and feasible (Shrestha et al., 2019; Xue et al., 2023).

### 4.2 Diversity hotspots for priority conservation of RCMPs

A total of 21 hotspot areas were identified in the analyses using all four thresholds, which are concentrated in the mountainous areas of southern and central China (**Figure 2**; **Supplementary Figure S3**), congruent with the hotspots reported in previous studies of specific target groups, such as endemic woody plants (Huang et al., 2012), endemic seed plants (Huang et al., 2016), threatened higher plants (Xue et al., 2023), and Chinese seed plants (Yang et al., 2022). This consistency indicates that RCMPs may play an important role in representing endemic, threatened, and economic plant attributes in hotspot identification and priority conservation efforts. (Chi et al., 2017) analyzed the distribution patterns and conservation status of threatened medicinal plants at the county level and compared their results with previous studies of medicinal plants. However, their analysis based on coarse county units of differing area failed to reliably and practically represent hotspots and conservation status to determine conservation priority. Although (Xia et al., 2022) used a fine grid of 50-km resolution, they employed only two algorithms (species richness and complementarity) and the top 5% threshold to analyze medicinal plant distribution patterns, overlooking newer hotspots accounted for by the weighted endemism algorithm and higher thresholds. In comparison to previous studies, we identified new hotspots, including Qilian Mountains, Qinling-Bashan Mountains, and southeastern Xizang, which have not been recognized in previous research. Additionally, eastern Qinling Mountains, Dabie Mountains, Yandang Mountains and Wuyi Mountains were identified as new hotspots under the top 10% and top 17% thresholds, while Tianshan Mountains, Changbai Mountains, and northern Taihang Mountains were identified as new hotspots using the top 30% criterion (**Supplementary Figure S3**). This improvement in hotspot identification can be attributed to the utilization of a higher resolution grid and consideration of multiple species attributes, algorithms, and biodiversity conservation target thresholds in hotspot identification.

Among the 21 hotspots identified, hotspots 1–12 were identified under all four thresholds and are the most effective protection areas. When the threshold was set to the top 10% and all species were included, a further five new hotspots (hotspots 13-17) were identified. Comprehensive protection should be prioritized in these important hotspot areas that contain all species, through the establishment of new nature reserves or national parks, long-term monitoring, and ensuring their inclusion in further protection efforts to achieve biodiversity conservation targets. When the threshold was set to the top 17%, conservation effectiveness was not significantly different from that for the top 10%, with the additional hotspots serving as a supplement to the top 10% hotspots, but a further 18 hotspots were identified. The top 30% threshold added hotspots 19, 20, and 21. Although these three hotspots were not identified at lower thresholds, they cannot be ignored because these hotspot areas remain important for certain species (Huang et al., 2022), such as *Cistanche deserticola* (EN, Xinjiang), *Panax ginseng* (CR, Changbai Mountains), *Taxus cuspidata* (EN, Changbai Mountains), *Ferula sinkiangensis* (CR, Xinjiang), and *Saussurea involucrata* (EN, Xinjiang).

### 4.3 Optimizing the layout of conservation networks for RCMPs

The conservation effectiveness of NNRs and PNRs in the top 5% of hotspots was 83.57% (**Table 1**), which is consistent with the findings of (Xia et al., 2022) for the conservation effectiveness of all medicinal plants at 83.3%. However, this study focuses on RCMPs, which are the most important medicinal plants with high economic value, including some threatened species. Our findings indicate that the current conservation network for RCMPs is insufficient, and conservation gaps remain. Although the conservation effectiveness of NNRs for the top 5% and top 10% of hotspots are higher than the effectiveness of PNRs, the conservation effectiveness of NNRs decreases more sharply with increasing hotspot threshold, resulting in lower conservation effectiveness for hotspots under the top 17% and top 30% thresholds (**Table 1**). This change in conservation effectiveness may be due to PNRs being approximately twice as numerous as NNRs (464 vs. 806), indicating that increasing the number of nature reserves may significantly improve conservation effectiveness and that PNRs act as an effective complement to NNRs in biodiversity conservation efforts. As NNRs generally have stricter management than PNRs and play a dominant role in biodiversity conservation (Zhao et al., 2013), NNRs should be further strengthened as the mainstay of biodiversity conservation. Meanwhile, the important role of PNRs in conservation should not be overlooked, and more attention needs to be paid to PNRs in the future. Moreover, numerous hotspots are distributed along provincial boundaries, such as the Sino-Vietnamese border, the junction of southern Guizhou and northwestern Guangxi, the Dalou-Wuling Mountains and the Wushan Mountains (**Figure 2**; **Supplementary Figure S3**). Eliminating the limitation that prevents nature reserves from crossing administrative boundaries in these areas may improve the integrity of habitats and the effectiveness of hotspot protection for medicinal plants (Xia et al., 2022), thereby enhancing RCMP conservation effectiveness. So, such studies could support optimizing the layout of current conservation networks under the first comprehensive national park system of China (Xu et al., 2017).

Analysis of conservation gaps shows that increasing the hotspot threshold led to more numerous apparent conservation gaps (from 16.43% to 29.85%) (**Figure 2**; **Supplementary Figure S4**). These unprotected areas are scattered throughout each hotspot, but are mainly found in southwest China, with an additional notable conservation gap in the hotspot areas of Xinjiang in the northwest (**Figure 2**). These conservation gaps were also identified for Chinese threatened plants (Zhang et al., 2015), threatened medicinal plants (Chi et al., 2017), and all medicinal plants (Xia et al., 2022), indicating that these conservation gaps have long standing and should be protected in future biodiversity conservation efforts through the establishment of new reserves to safeguard these hotspots of RCMP diversity. In addition, although all species are included in the top 10% of hotspots, large portions of RCMP populations and occurrence ranges lack effective conservation, and the Aichi targets have not yet been reached (Secretariat of the Convention on Biological Diversity, 2020). Therefore, to achieve the more ambitious post-2020 biodiversity conservation targets, the populations and distribution ranges of more species should receive effective conservation. In the numerous conservation gaps identified among nature reserves, which are mostly located in Yunnan, Xinjiang, Guizhou, central Hunan, west Guangdong, and south Jiangxi (**Figure 2**), more nature reserves or protected areas should be established as soon as possible.

At the species level, 13 species, including *Ferula fukanensis* and *Cistanche tubulosa*, the protection ratio for their distribution records are < 30% (post-2020 biodiversity conservation targets) or even < 17% (Aichi targets) (**Supplementary Table S1**; **Supplementary Figure S6**). Focusing on those species, prompt implementation of priority conservation planning measures, such as the establishment of conservation areas, micro-reserves, or *in-situ* protection measures is urgently needed. Additionally, given that some important and commonly used RCMPs have not gained widespread attention, including *Panax ginseng*, *Cistanche deserticola*, *Fritillaria thunbergii*, and *Fritillaria walujewi* (Huang et al., 2022), special attention should be paid to these important medicinal species in future conservation management efforts. It worth mentioned than currently most medicinal plant involved in this study are listed in the National Key Protected Wild Plants in China, which may relieve the RCMP extinction risk (Xue et al., 2023; Huang et al., 2022).

### 4.4 RCMP conservation measures under climate change scenarios

The trends in potential suitable habitat areas for RCMPs indicate decreases in species richness in central and southeastern China, while species richness is expected to increase in southeast Xizang and the central Himalayas under the RCP 2.6 climate scenario (**Figures 3A, C**). In comparison, under RCP 8.5 climate scenario (**Figures 3B, F**), more dramatic decreases in species richness are predicted in central and southeastern China and the affected area is expanded, while species richness increases markedly in the central Himalayas. Overall, a significant shift in potential suitable habitat areas from east to west is expected. Similar trends have been observed in previous studies on gymnosperms (Li et al., 2021), which may be due to the west-to-east high-to-low trend of China’s topography, as the high-elevation regions in the western part of the country may experience a shift from cold to warm climate, satisfying the habitat needs of species originally distributed in eastern regions, while the high environmental heterogeneity of mountainous areas in the west provides refuges for the survival of certain species during future climate changes (Tang et al., 2006; Chen et al., 2011; Tang et al., 2018). In response to a changing climate, regions with relatively stable potential suitable habitat areas and regions with increasing species richness should be considered for priority conservation (Li et al., 2021) through the establishment of long-term *in-situ* conservation areas. Such regions include the Qilian Mountains, southeast Xizang, and the central Himalayas. For areas with dramatic losses of species richness predicted, such as Qinling-Bashan, the northern Hengduan Mountains, Xishuangbanna, the Nanling Mountains, Dalou-Wuling Mountains, and Wuyi Mountains, dynamic monitoring, *ex-situ* and *in-situ* conservation efforts, and relevant policies should be implemented immediately to prevent the local extinction of species and loss of genetic diversity.

Our results show that significant losses of habitat area for many species will occur in the future under either climate scenario - RCP 2.6 or RCP 8.5. Compared to the RCP 2.6 scenario, we found that more species will lose potential suitable habitat under RCP 8.5, with 205 RCMPs (vs. 125 RCMPs under RCP 2.6) losing more than 10% of their potential suitable habitat area and 151 RCMPs (vs. 52 RCMPs under RCP 2.6) losing more than 30% potential of their suitable habitat area (**Supplementary Figure S8**; **Supplementary Table S3**). These findings indicate that climate change will pose a serious threat to the survival of RCMPs by altering their occurrence ranges. Furthermore, compared to the RCP 2.6 climate scenario, more unthreatened RCMPs (72 vs. 18) will be upgraded to threatened categories under the RCP 8.5 climate scenario (**Table 2**), posing a huge challenge to conservation efforts for RCMPs. Notably, two species, *Stephania mashanica* (EN) and *Illicium difengpi* (EN), are predicted to lose all their potential suitable habitat areas, leading to extinction under the RCP 8.5 climate scenario. Meanwhile, we found that 186 and 241 RCMPs will expand their suitable habitat areas under the RCP 2.6 and RCP 8.5 scenarios, respectively (**Figures 3A, B**; **Supplementary Table S3**). However, these species are mostly distributed in the Hengduan Mountains in southern Guizhou, which is densely populated and has high levels of human activity (Zhang et al., 2021; Su et al., 2020), which limits the dispersal of these species, preventing them from fully occupying their suitable habitat area. Therefore, for species with reductions in their potential suitable habitat area and upgraded threatened categories predicted, conservation efforts must be implemented immediately, including *in-situ*, *ex-situ*, and near-situ conservation measures, as well as vigorous artificial cultivation efforts. To reduce the damage to wild populations of these species, strategies to counteract the trend of increasing threat to RCMPs from multiple perspectives are needed in response to climate change.

### 4.5 Establishment of a comprehensive priority conservation framework for RCMPs

**Figure 4:**
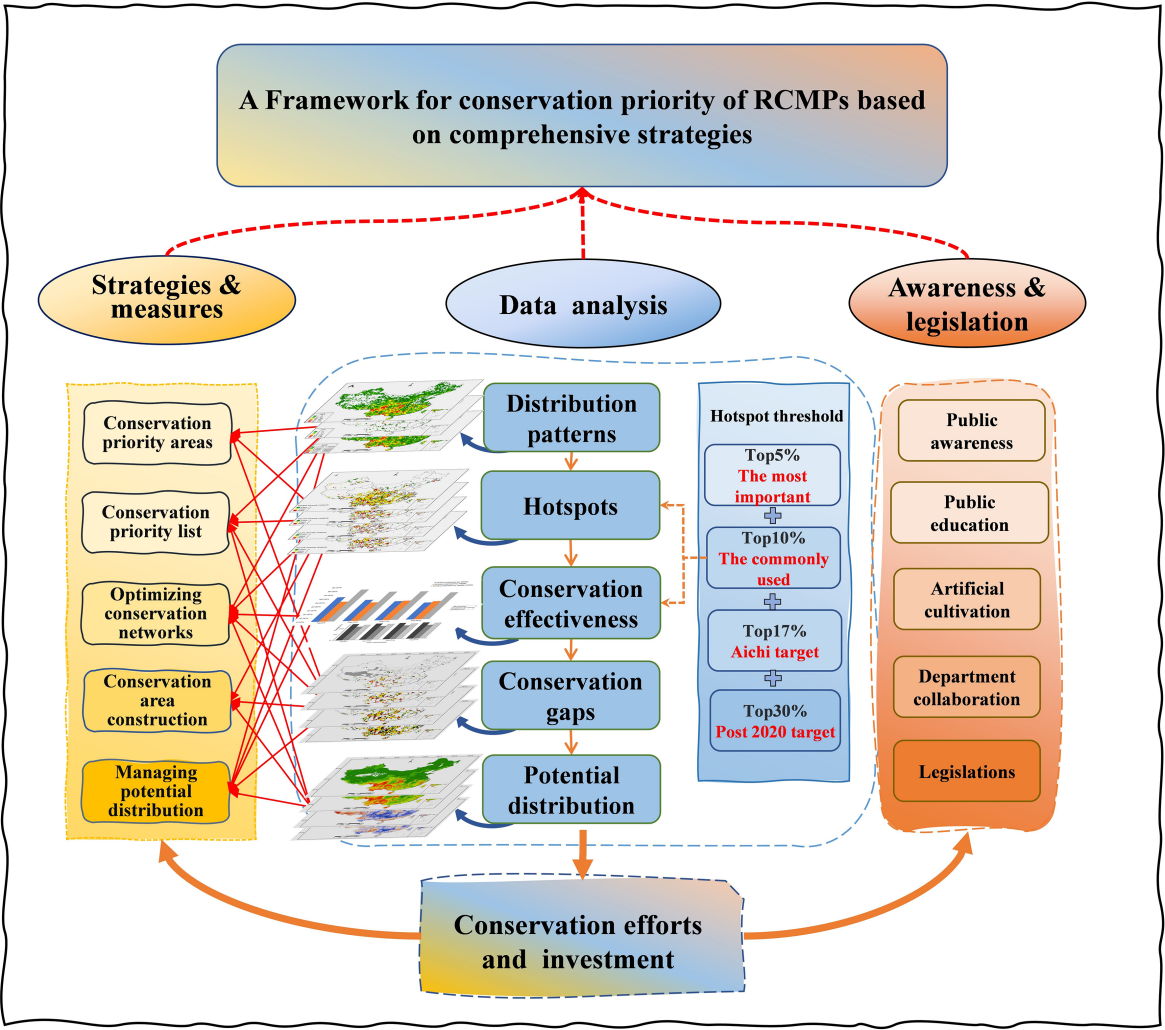
A framework for conservation priority of RCMPs based on comprehensive strategies.

Based on high-precision species distribution data, this study explored five aspects of RCMPs conservation: the geographic distribution pattern of REMPs hotspots at different thresholds, conservation effectiveness and gaps in the current conservation network, and the impacts of climate change on potential distribution areas. These findings provide solid data to support the establishment of a prioritized conservation management framework for RCMPs. Three algorithms were used to study the geographic distribution pattern of RCMPs, reflecting the spatial distribution characteristics of species richness, complementarity, and narrow endemism. Using these algorithms in combination with correlation analysis of distribution patterns, this study provides support for targeted conservation of areas with high species richness, regionally representative species, and species that exhibit narrow endemism, while also elucidating the conservation value of various regions to biodiversity. Second, the hotspots identified using different thresholds provide reference data for prioritization of conservation management areas for RCMPs and support the development of a prioritized conservation list for RCMPs. In future biodiversity conservation works, reference to the diversity hotspots identified at various thresholds in this study can help achieve the Aichi and post-2020 biodiversity conservation framework targets. For example, the top 5% and top 10% hotspots include all medicinal plant species. Because these medicinal plants are threatened, have high medicinal value, and face great extinction pressure, we propose comprehensive protection for hotspots within the top 10%. The top 17% hotspots further strengthen the protection provided by the top 10% hotspots and represent an Aichi target that can be achieved with minimal protection effort. The hotspots identified in the top 30% include all biodiversity hotspots identified at the three other thresholds, as well as three additional hotspots, which are also post-2020 biodiversity conservation framework targets that require consideration in future conservation work. Furthermore, through comprehensive evaluation of the conservation effectiveness and gaps of the current conservation network for hotspots at various thresholds in combination with changes in the reference ranges, this study provides support for prioritizing the layout of the biodiversity conservation network. For example, our findings suggest establishing new nature reserves or micro-reserves in areas with many protection gaps, and conducting *in-situ* protection, *ex-situ* protection, and near-situ protection in areas with species increases or losses. Finally, based on various strategies and measures for conservation of prioritized areas, including prioritized conservation lists, optimized conservation networks, and management of potential distribution areas, this study emphasizes the importance of public awareness, data sharing and exchange, departmental cooperation, and incentive mechanisms in the development of a comprehensive framework for prioritized conservation of RCMPs (**Figure 4**).

## 5 Conclusion

Plants listed in the RCMPs are characterized by the combination of significant medicinal value and clear threats to their wild populations. This study used a high-resolution grid, precise species distribution data, and three algorithms to determine the geographic distribution patterns of RCMPs. We identified diversity hotspots of RCMPs using multiple hotspot thresholds, analyzed their conservation effectiveness, and predicted potential distribution areas, providing new insights into the biogeography and conservation of RCMPs. The geographic distribution patterns of RCMPs varied depending on the algorithm used, providing targeted protection goals for various types of species. The hotspots identified in this study are located mainly in the southwestern region of China, with a few in the northern region. Additionally, new hotspots were added to the list of traditional hotspots, including the Qilian Mountains, Bashan-Qinling, and southeast Xizang, using the complementarity and weighted endemism algorithms. Based on conservation effectiveness and gap analysis, at least 16.43% of hotspot grid cells contained no natural reserves, covering at least 70.96% of RCMPs. Changes in hotspot areas and conservation gaps were determined under various hotspot thresholds. Overall, significant conservation gaps were found in areas such as the Hengduan Mountains, Guizhou, and Xinjiang, which require the establishment of new nature reserves or specialized botanical gardens in the future. Targeted measures, such as *in-situ* protection, near-situ protection, and *ex-situ* protection, should be implemented in other areas with conservation gaps or adjacent hotspots. Furthermore, areas such as the Hengduan Mountains, southeast Xizang and the central Himalayas are likely to gain species in the future and may become long-term priority protection areas for RCMPs.

## Data Availability

The original contributions presented in the study are publicly available. This data can be found here: https://doi.org/10.6084/m9.figshare.30563810.
