# Supplementary material for: Distribution patterns, conservation status and suitable habitat areas of highly valuable medicinal plants in China
Source: Front Plant Sci. 2025 Dec 3;16:1657997. doi: 10.3389/fpls.2025.1657997 (PMC12708912; doi:10.3389/fpls.2025.1657997)
Supplement: Supplementary file 2 [file DataSheet1.pdf]

Supplementary Figures

(Figures S1-S8)

Distribution patterns, conservation status and suitable habitat areas of highly valuable medicinal plants in China

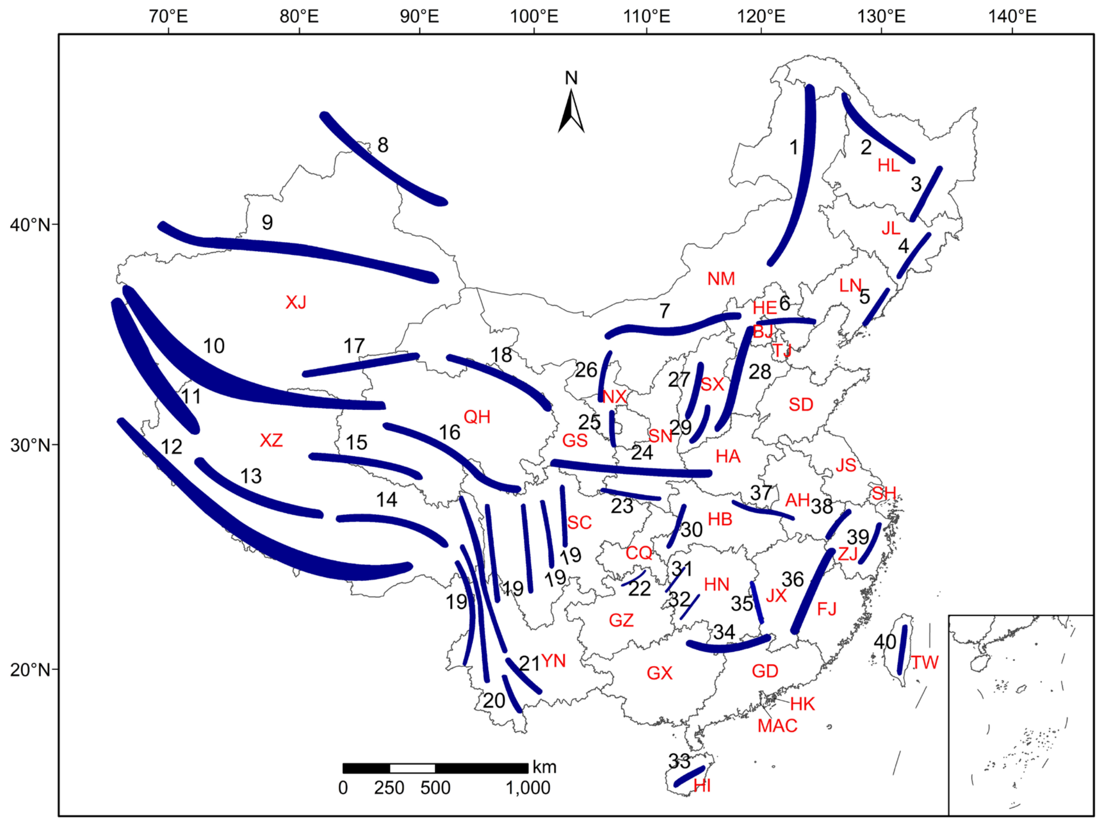

FIGURE S1 Map of the main mountain ranges and administrative divisions in China: 1. Daxing'anling Mountains, 2. Xiaoxing'anling Mountains, 3. Changbai Mountains, 4. Zhangguangcailing Mountains, 5. Longgang Mountains, 6. Yanshan Mountains, 7. Yinshan Mountains, 8. Altai Mountains 9. Tianshan Mountains, 10. Kunlun Mountains, 11. Karakorum Mountains, 12. Himalayas, 13. Gangdisi Mountains, 14. Nyainqntanglha Mountains, 15. Danggula Mountains, 16. Bayankala Mountains, 17. Aerjin Mountains, 18. Qilian Mountains, 19Hengduan Mountains, 20. Wuliang Mountains, 21. Ailao Mountains, 22. Dalou Mountains, 23. Bashan Mountains, 24.

Qinling Mountains, 25. Liupan Mountains, 26. Helan Mountains, 27. Luliang Mountains, 28. Taihang Mountains, 29. Zhongtiao Mountains, 30. Wushan Mountains, 31. Wuling Mountains, 32 Xuefeng Mountains, 33. Wuzhi Mountains, 34. Nanling Mountains, 35. Luoxiao Mountains, 36. Wuyi Mountains, 37. Dabie Mountains, 38. Tianmu Mountains, 39. Yandang Mountains, 40. Yushan Mountains (Wang et al., 2004). The red characters represent China's administrative divisions: Heilongjiang (HL), Jilin (JL), Liaoning (LN), Neimeng (NM), Hebei (HE), Beijing (BJ), Tianjing (TJ), Xinjiang (XJ), Xizang (XZ), Qinghai (QH), Gansu (GS), Ningxia (NX), Shaanxi (SN), Shanxi (SX), Shandong (SD), Henan (HA), Sichuan (SC), Chongqing (CQ), Hubei (HB), Anhui (AH), Jiangsu (JS), Shanghai (SH), Yunnan (YN), Guizhou (GZ), Hunan (HN), Jiangxi (JX), Zhejiang (ZJ), Fujian (FJ), Guangxi (GX), Guangdong (GD), Taiwan (TW), Hong Kong (HK), Macau (MAC), and Hainan (HN).

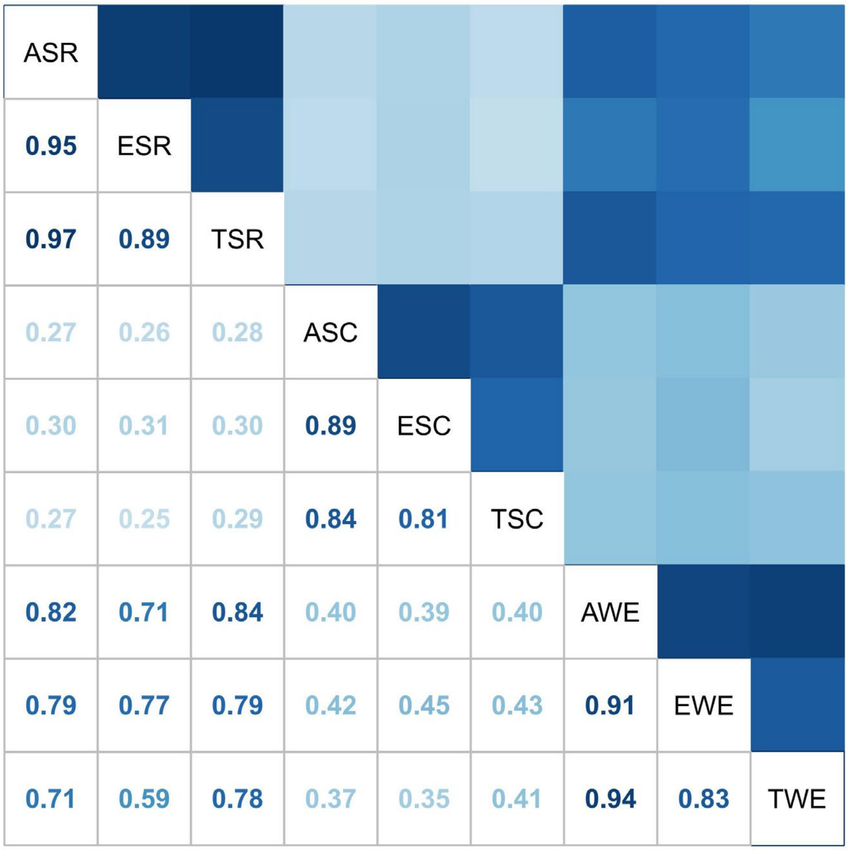

FIGURE S2 Correlogram of three algorithms described in the *Red Data Book of Chinese Medicinal Plants* (RCMPs). Distribution patterns of All RCMPs (ASR), Endemic RCMPs (ESR), and Threatened RCMPs (TSR) based on species richness. Distribution patterns of All RCMPs (ASC), Endemic RCMPs (ESC), and Threatened RCMPs (TSC) based on species complementarity. Distribution patterns of All RCMPs (AWE), Endemic RCMPs (EWE), and Threatened RCMPs (TWE) based on weighted endemism. All correlation coefficients are significant at $P < 0.01$.

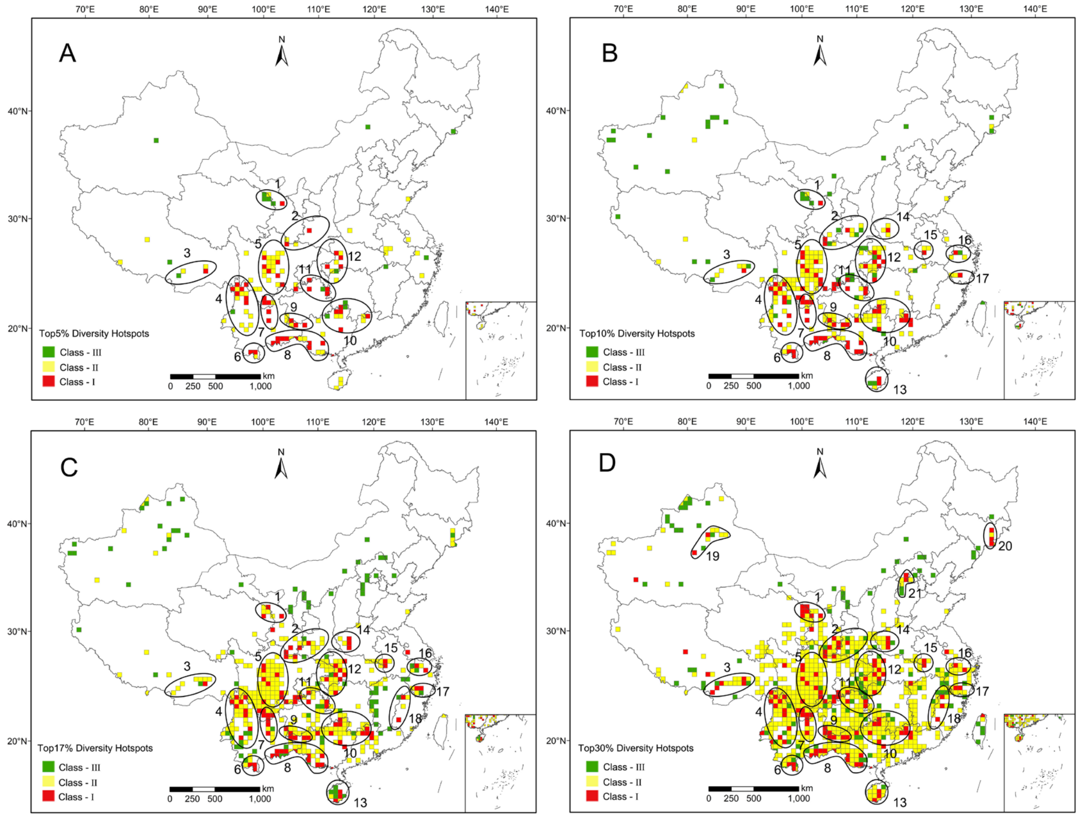

FIGURE S3 Final diversity hotspots of RCMPs at thresholds of the A) Top 5%, B) Top 10%, C) Top 17%, and D) Top 30%. Black circles indicate 21 diversity hotspot areas: (1) Qilian Mountains, (2) Qinling-Bashan Mountains, (3) southeast Xizang, (4) southern Hengduan Mountains, (5) northern Hengduan Mountains, (6) Xishuangbanna, (7) northern Yunnan, (8) Sino-Vietnamese border, (9) the junction of southern Guizhou and northwestern Guangxi, (10) Nanling Mountains, (11) Dalou-Wuling Mountains, (12) Wushan Mountains, (13) Hainan Island, (14) eastern Qinling Mountains, (15) Dabie Mountains, (16) Tianmu Mountain, (17) Yandang Mountain, (18) Wuyi Mountains, (19) Tianshan Mountains, (20) Changbai Mountains, and (21) northern Taihang Mountains.

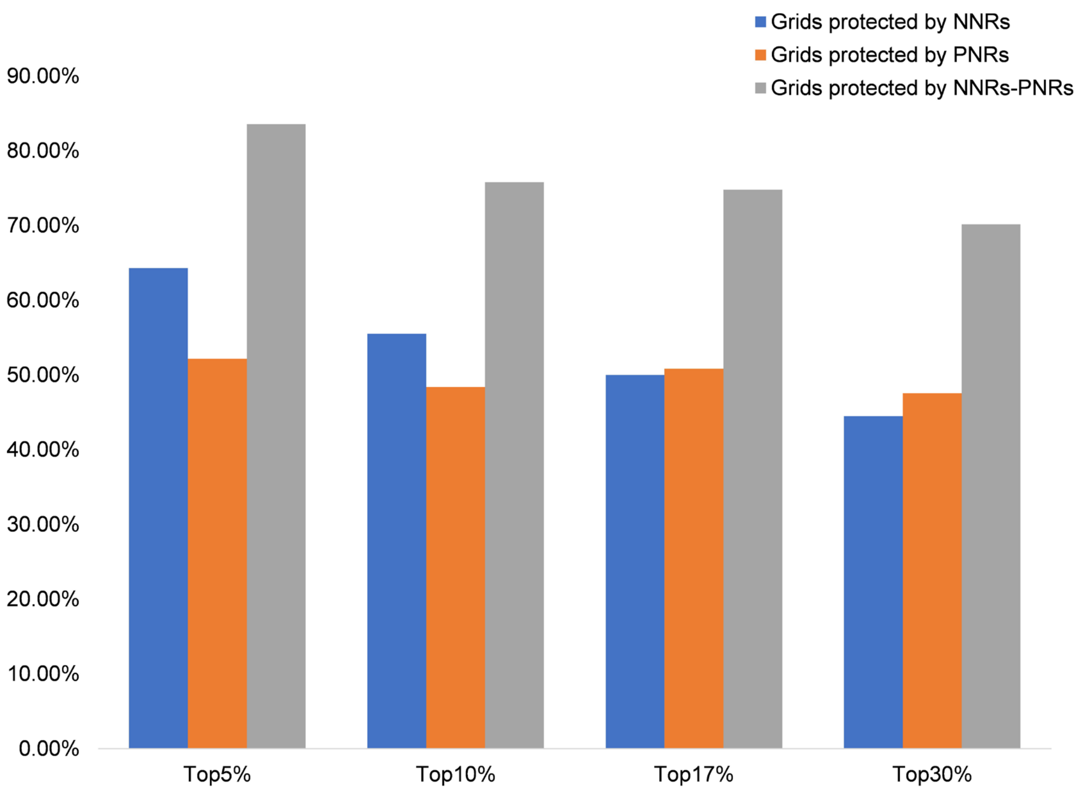

FIGURE S4 Conservation effectiveness of national nature reserves (NNRs) and provincial nature reserves (PNRs) for hotspots at four thresholds.

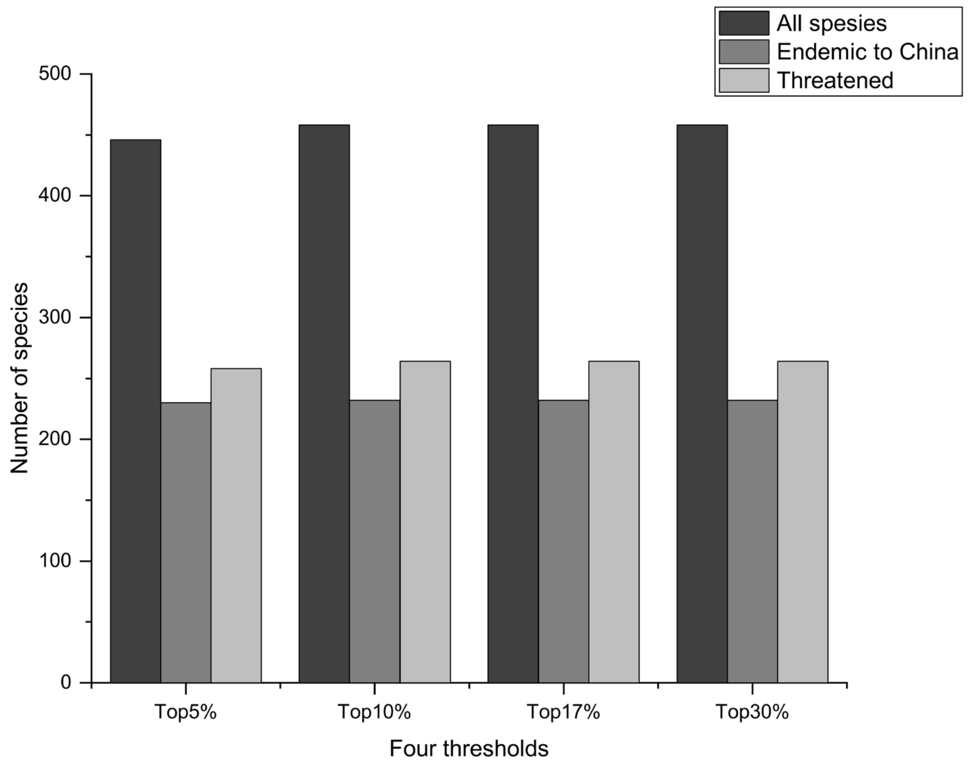

FIGURE S5 Conservation effectiveness for various biological attributes under four hotspot thresholds.

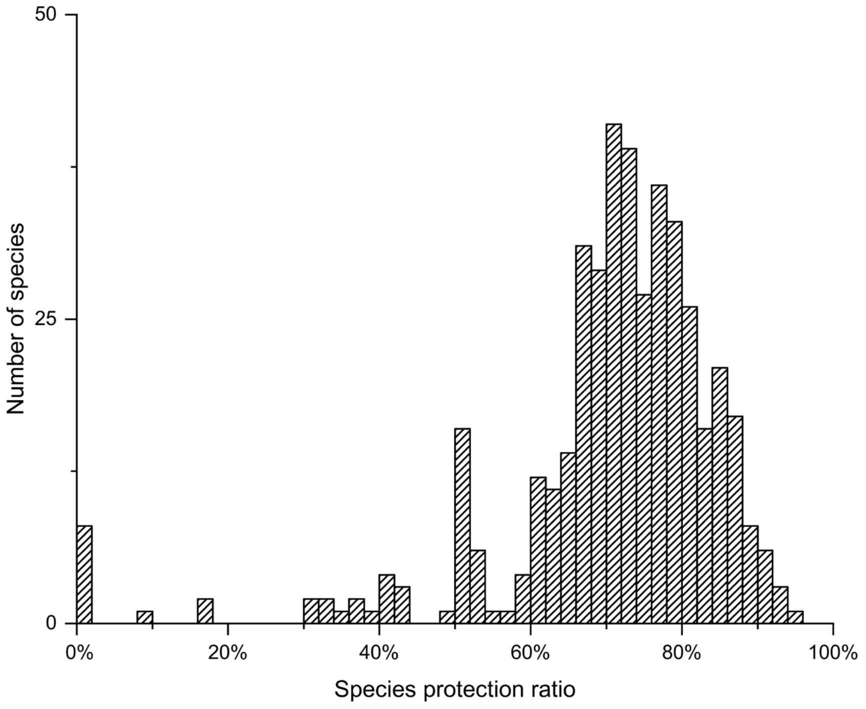

FIGURE S6 RCMPs species distribution site protection ratios.

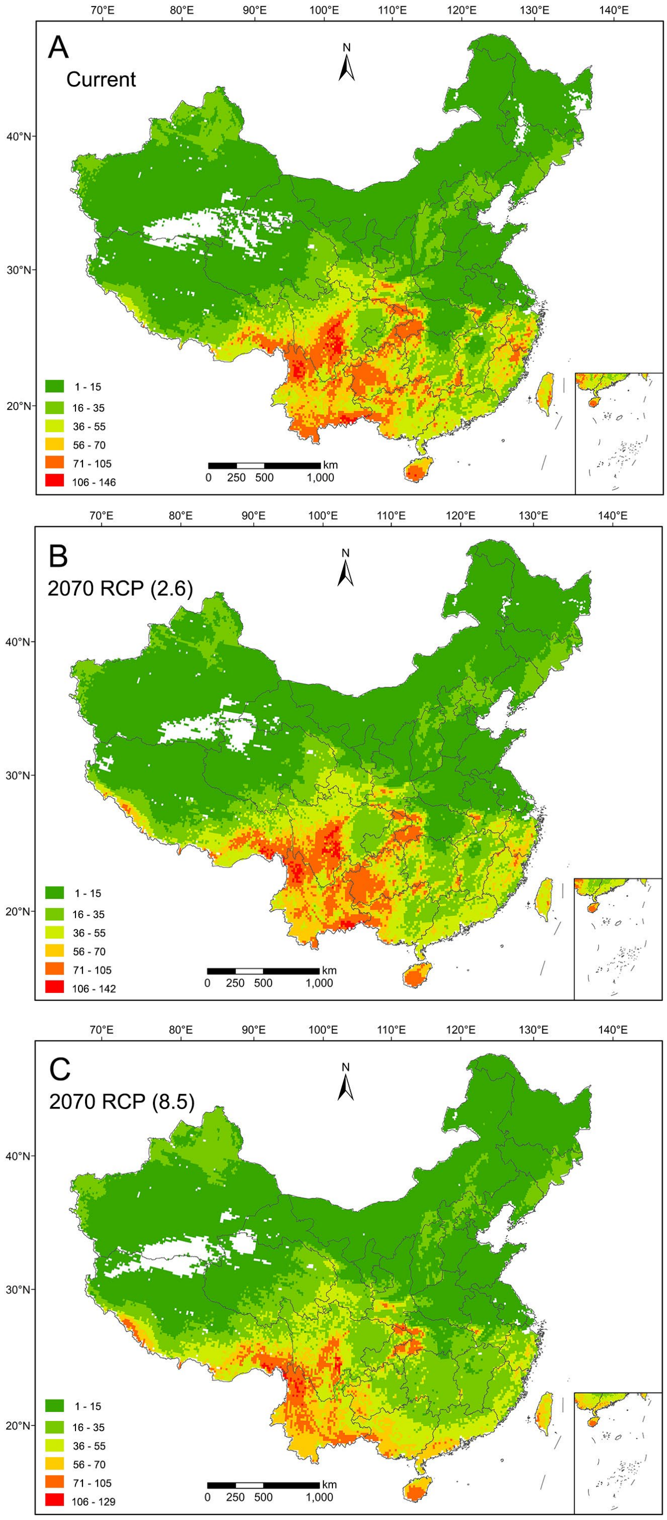

FIGURE S7 Distribution patterns of species richness of 1959 RCMPs based on the results of MaxEnt. Time periods: recent past (1960–1990) and future (2070). Emission scenarios: representative concentration pathway (RCP) 2.6 and RCP 8.5.

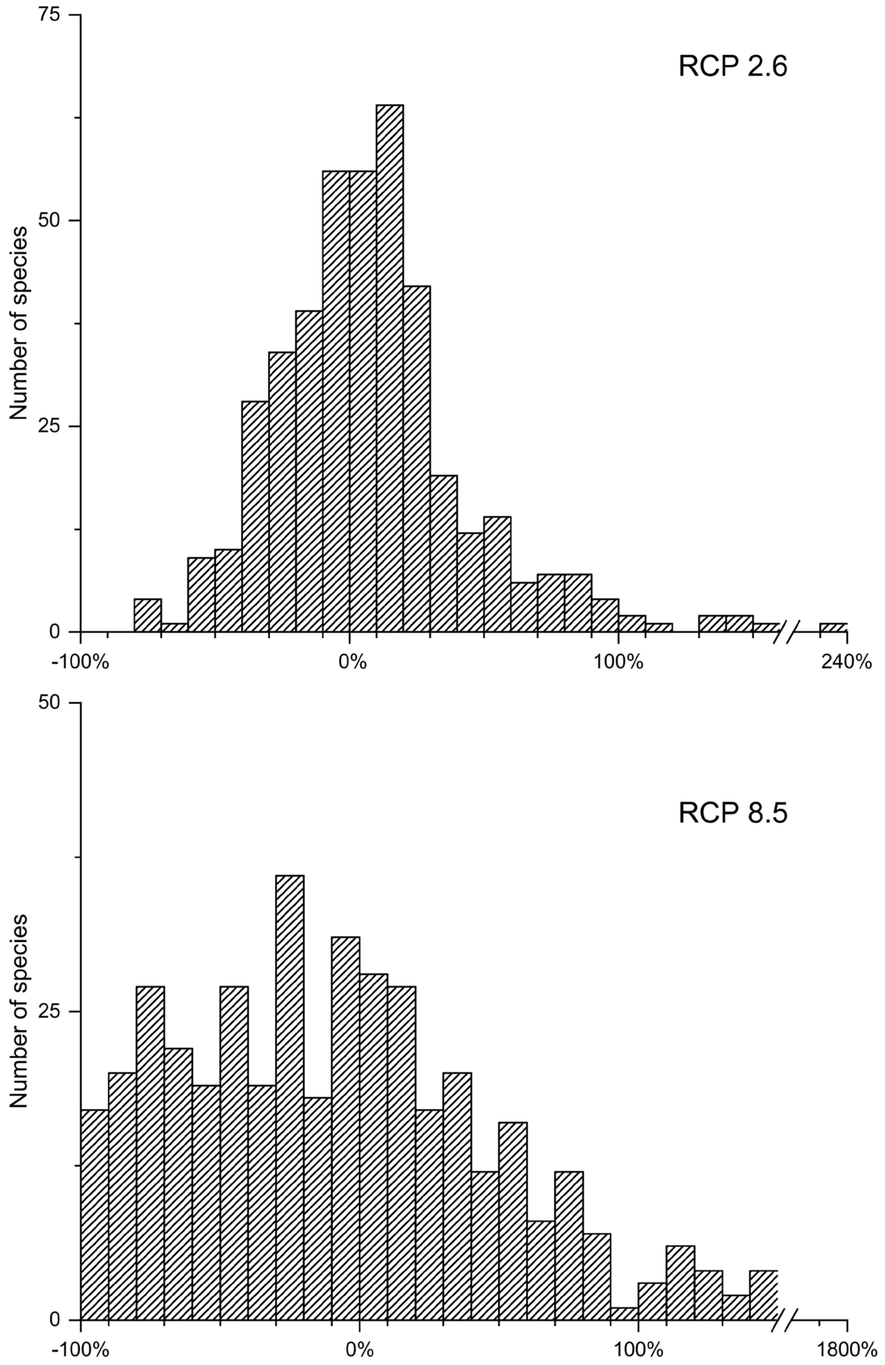

FIGURE S8 Changes in range sizes of threatened higher plants in China under two emission scenarios, representative concentration pathway (RCP) 2.6 and RCP 8.5.
